# Antimicrobial Activity of MgB_2_ Powders Produced via Reactive Liquid Infiltration Method

**DOI:** 10.3390/molecules26164966

**Published:** 2021-08-17

**Authors:** Santanu Kumar Padhi, Nicoletta Baglieri, Valentina Bonino, Angelo Agostino, Lorenza Operti, Nicolae Dan Batalu, Mariana Carmen Chifiriuc, Marcela Popa, Mihail Burdusel, Mihai Alexandru Grigoroscuta, Gheorghe Virgil Aldica, Dana Radu, Petre Badica, Marco Truccato

**Affiliations:** 1 Physics and Chemistry Departments, University of Turin, Via P. Giuria 1-7, 10125 Turin, Italy; santanukumar.padhi@unito.it (S.K.P.); nicoletta.baglieri@edu.unito.it (N.B.); valentina.bonino@unito.it (V.B.); angelo.agostino@unito.it (A.A.); lorenza.operti@unito.it (L.O.); 2 European Synchrotron Radiation Facility, 71 Avenue des Martyrs, 38000 Grenoble, France; 3 Metallic Materials Science, Physical Metallurgy Department, Faculty of Materials Science and Engineering, University Politehnica of Bucharest, Splaiul Independentei 313, 060042 Bucharest, Romania; dan.batalu@upb.ro; 4 Faculty of Biology, Research Institute of the University of Bucharest (ICUB), University of Bucharest, Spl. Independentei 91-95, 050095 Bucharest, Romania; carmen.chifiriuc@bio.unibuc.ro (M.C.C.); bmarcelica@yahoo.com (M.P.); 5 Academy of Romanian Scientists, 050094 Bucharest, Romania; 6 National Institute of Materials Physics, Street Atomistilor 405A, 077125 Magurele, Romania; mihaita_burdusel@yahoo.com (M.B.); alex_bebe07@yahoo.com (M.A.G.); aldica2000@yahoo.com (G.V.A.); dana.radu@infim.ro (D.R.)

**Keywords:** MgB_2_, reactive liquid infiltration method, antimicrobial activity, biofilms

## Abstract

We report for the first time on the antimicrobial activity of MgB_2_ powders produced via the Reactive Liquid Infiltration (RLI) process. Samples with MgB_2_ wt.% ranging from 2% to 99% were obtained and characterized, observing different levels of grain aggregation and of impurity phases. Their antimicrobial activity was tested against *Staphylococcus aureus* ATCC BAA 1026, *Enterococcus faecalis* ATCC 29212, *Escherichia coli* ATCC 25922, *Pseudomonas aeruginosa* ATCC 27853, and *Candida albicans* ATCC 10231. A general correlation is observed between the antibacterial activity and the MgB_2_ wt.%, but the sample microstructure also appears to be very important. RLI-MgB_2_ powders show better performances compared to commercial powders against microbial strains in the planktonic form, and their activity against biofilms is also very similar.

## 1. Introduction

Since the discovery of its superconducting properties [1], MgB_2_ has attracted a lot of interest both from the theoretical [2,3,4], and from the practical point of view, with many applications that have been explored and sometimes commercially developed and delivered to the market [5,6,7,8,9,10]. Preparation methods for this material span a large variety of different techniques [11,12,13,14,15,16,17,18,19,20,21,22,23], and among them the Reactive Liquid Infiltration (RLI) method has proved to be suitable to produce objects with complex shapes, high density and good superconducting characteristics [24,25,26]. More recently, new interest in MgB_2_ has been sparked by its unexpected application to the completely different field of biomaterials, which started with the first report by Batalu et al. [27]. For instance, MgB_2_ in the form of nanosheets has shown promising results about the possibility to induce hydrogen release at targeted gastric cancer cells, paving the way for a novel hydrogenochemotherapy of digestive tumors that is expected to have high efficacy and reduced toxic side effects compared to ordinary chemotherapy [28]. MgB_2_ nanosheets have also proved to have a good osteogenic potential for bone-disease-related therapeutics since they are able to enhance the osteoblast differentiation of mouse mesenchymal stem cells when embedded in polymeric scaffolds [29].

In the specific field of antimicrobial materials, it has been shown that the growth of biofilms formed by the *Escherichia coli* Gram-negative strain is strongly inhibited on MgB_2_ substrates. Although the specific mechanisms of this antibiofilm activity are not completely elucidated, the inhibitory effect can be at least partially explained by the interference with the adherence mechanism [27]. On the one hand, this phenomenon is correlated with the pH increase due to the decomposition reaction MgB_2_ + 2 H_2_O → Mg(OH)_2_ + 2B + H_2_ taking place at the material surface in physiological solution, with corresponding hydrogen release and basification, which is able to affect the physico-chemical properties of the cellular wall and to decrease its interaction with the adherence surface [30,31,32,33,34]. On the other hand, it is known that an excessive B amount could interfere with the quorum sensing mechanism that is responsible for the regulation of some phenotypes (such as biofilm formation, motility, and expression of virulence factors), all of them depending on the coordination of the bacterial behavior at the population level in a density-dependent manner [35,36]. Additionally, from the point of view of MgB_2_ interaction with tumor cells (HeLa and HT-29 lines), it has been recently demonstrated that this material can induce apoptosis and arrests the tumor cells in the S phase, suggesting an interference with the DNA synthesis and, consequently, with the cellular proliferation [37]. Whatever the mechanisms are at the cellular level, the anti-infectious action of MgB_2_ has also been revealed in vivo, where a significant decrease in the pathogenic *Escherichia coli* charge in liver, spleen, and peritoneal liquid has been detected after treatments of the infected mice with this material [37,38]. Finally, MgB_2_ powders have been shown to be effective also against planktonic and biofilm fungi cells involved in the bio-deterioration of heritage buildings and objects [39]. In view of future applications, MgB_2_-polyvinylpyrrolidone (MgB_2_–PVP) composite materials have also been fabricated and tested, showing that they possess a good inhibitory activity on the planktonic bacterial growth, even if this effect is less apparent and strain-specific in the case of biofilms [38]. In any case, all of these recent investigations of the MgB_2_ antimicrobial activity have been carried out only by using commercial powders as raw materials, available from major chemical companies. No investigation has been performed so far on the antimicrobial activity of MgB_2_ materials produced by means of the RLI method, which is of great interest because of its potential to be manufactured in complex shapes, and, consequently, in self-sterilizing medical instruments. The present paper is specifically dedicated to fill this knowledge gap by assessing the physico-chemical and the bioactivity properties of MgB_2_ samples produced via RLI.

## 2. Experimental

Samples have been produced by using as precursors an amorphous B powder (Sigma Aldrich, Taufkirchen, Germany, nominal purity > 95%, particle size ≤ 1 µm) and Mg granules (Alfa Aesar, Kandel, Germany, nominal purity 99.8%, 1.70 mm). These precursors were placed inside a degassed steel container one after the other in an alternate configuration. Then, the ends of the precursor-filled steel container were sealed by hydraulic pressing.

Different Mg:B stoichiometric ratios have been tested, with values increasing from 1.4:2 to 1.8:2. The thermal cycles for material synthesis consisted in heating the sealed container in a quartz furnace up to a temperature ranging from 670 °C to 800 °C and dwelling at this temperature for various durations ranging from 2 to 6 h. Heating ramps were performed at a rate of 5 °C/min, and an intermediate plateau at 150 °C (1 h dwell) was maintained before reaching the final dwelling temperature.

During the synthesis process, a constant Ar flux of 0.2 L/min was maintained. Before the synthesis cycle, the steel reaction chamber was degassed at 200 °C for 7 h with a 0.2 L/min Ar flux and stored in a glove box. The precursor and steel tube handling was done inside the glove box to reduce contamination by oxygen.

X-ray diffraction (XRD) patterns of the samples were acquired with a Philips X’Pert diffractometer (PW3040/60 X’Pert PRO model). The absolute scan mode for a 2θ range between 10° and 110° was used with a step size of 0.02°. Microstructural and elemental analysis was carried out by means of scanning electron microscopy (SEM, Lyra 3XMU/Tescan), equipped with a Peltier cooled Bruker Quantax 200 X-ray detector for energy-dispersive X-ray spectroscopy (EDX). Simultaneous thermogravimetry and differential scanning calorimetry (TG/DSC) analyses were performed with an SDT Q600 model (TA Instruments). Powder samples of (20 ± 0.2) mg were placed in alumina cups while keeping an airflow of 50 cm^3^/min. The TG/DSC measurements were carried out in air at a heating rate of 10 °C/min from ambient temperature to 1050 °C. For the DSC exothermal events, the peak upwards convention was used.

Antimicrobial activity of the MgB_2_ powders was tested against four bacterial strains, both Gram-positive (*Staphylococcus aureus* ATCC BAA 1026, *Enterococcus faecalis* ATCC 29212) and Gram-negative (*Escherichia coli* ATCC 25922, and *Pseudomonas aeruginosa* ATCC 27853) and one yeast strain (*Candida albicans* ATCC 10231) by microdilution method, using a range of binary dilutions prepared for each powder from a stock solution of 10 mg/mL in dimethyl sulfoxide (DMSO).

Minimal Inhibitory Concentration (MIC) of the tested MgB_2_ solutions was determined as the lowest concentration that prevented visible growth of microorganisms. The assay was performed in 96-well plates by dilution with Triptone Soy Broth (TSB). A 10^5^ CFU/mL (Colony Forming Units) bacterial suspension was added to each dilution and incubated at 37 °C for 24 h. For the yeast strain (*Candida albicans*) a 10^7^ CFU/mL suspension was used. DMSO was also tested as a reference. Microbial concentration was controlled by reading the optical density of each dilution at 620 nm.

The Minimal Biofilm Inhibitory Concentration (MBIC) was also evaluated for the MgB_2_ powders. After determining the MIC, the content of the 96-multiwell plates was discarded and the plates were washed with sterile phosphate-buffered saline (PBS; pH 7.2), fixed with methanol, stained with aqueous crystal violet 1% solution, and decolored with acetic acid 33%. The optical density of each well stained with crystal violet was measured at 495 nm using a microtiter-plate reader (EZ Reader 400, Biochrom).

## 3. Results and Discussion

**Figure 1:**
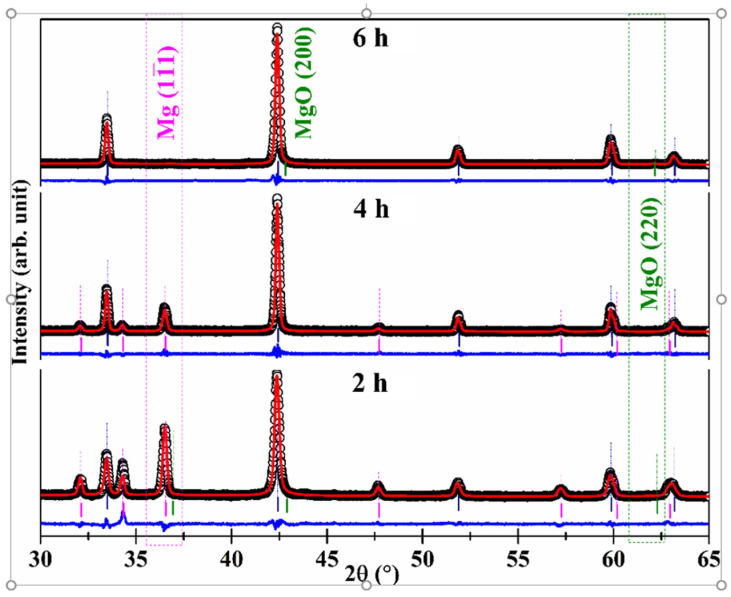
Representative XRD patterns of MgB_2_ samples obtained with Mg:B = 1.4:2 at *T* = 800 °C and corresponding to increasing processing durations of 2 h (sample MBNB16), 4 h (MBNB04) and 6 h (MBNB06), respectively. Black void circles represent experimental data points, red solid lines are the best fits, and solid blue lines are the corresponding residuals. The expected positions of the peaks are labeled by vertical solid ticks in magenta (Mg), olive (MgO), and navy (MgB_2_) colors, respectively. Expected positions of peaks Mg$\left(1\bar{1}1\right)$ and MgO (220) are enclosed in magenta and olive dashed boxes to guide the eye.

In Figure 1, both the observed XRD patterns and the calculated patterns generated after Rietveld refinement are presented for some samples prepared at *T* = 800 °C with the stoichiometric ratio Mg:B = 1.4:2. The Rietveld refinements were performed using the MAUD (Material Analysis Using Diffraction) program [40,41]. In Figure 1, some vertical lines indicating the positions of the different peaks and the residuals of the fitting procedures are also shown for clarity. The percentage weight fractions (wt.%) of the different crystalline phases resulting from the refinement procedures are reported in Table 1 for all of the samples, along with the standard R-factor values (i.e., the weighted profile R-factor *R_wp_* and the expected R-factor *R_exp_*) characterizing the quality of the fits. Table 1 also reports the main synthesis parameters of the samples.

It can be noticed that the quality of the refinements is generally good and that MgB_2_ always represents the major phase, as expected, apart from the case of the sample that was synthesized at 670 °C (MBNB01). In this special case, by far the most dominant phase is represented by Mg_2_B_25_, which is an intermediate compound in the reaction leading to the formation of MgB_2_ [42,43]. This result confirms the favorable kinetics for the production of Mg_2_B_25_ at this temperature, whereas, for a significant rate of the reaction 21 Mg (*l*) + 2 Mg_2_B_25_ (*s*)→25 MgB_2_ (*s*) to take place, temperatures above 750 °C are necessary [42]. This sample was intended to test the antibacterial activity of the Mg_2_B_25_ precursor phase. For all of the other samples, only Mg and MgO were detected as minor components, with maximum percentage amounts of 29 and 5 wt.%, respectively.

**Figure 2:**
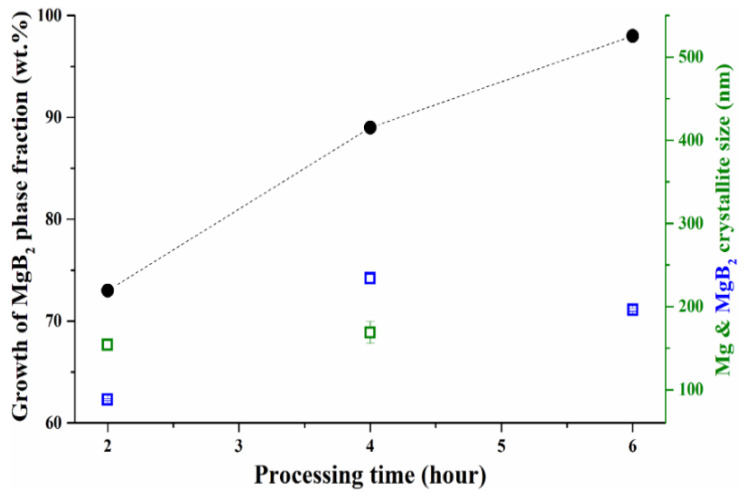
Evolution of the MgB_2_ production versus synthesis duration for *T* = 800 °C and Mg:B stoichiometric ratio equal to 1.4:2. Black solid circles represent the MgB_2_ percentage weight fractions, and green and blue void squares are the Mg and MgB_2_ crystallite sizes, respectively.

Figure 2 summarizes the results of the Rietveld refinements for the samples shown in Figure 1. It can be noticed that the MgB_2_ weight fraction increases with the synthesis duration up to a maximum of 99 wt.%, whereas no clear trend can be detected in the crystallite sizes, whose values span from about 80 to 240 nm. The latter observation was somewhat expected, since the reliability of crystallite size estimation significantly decreases for values greater than 100–150 nm.

**Figure 3:**
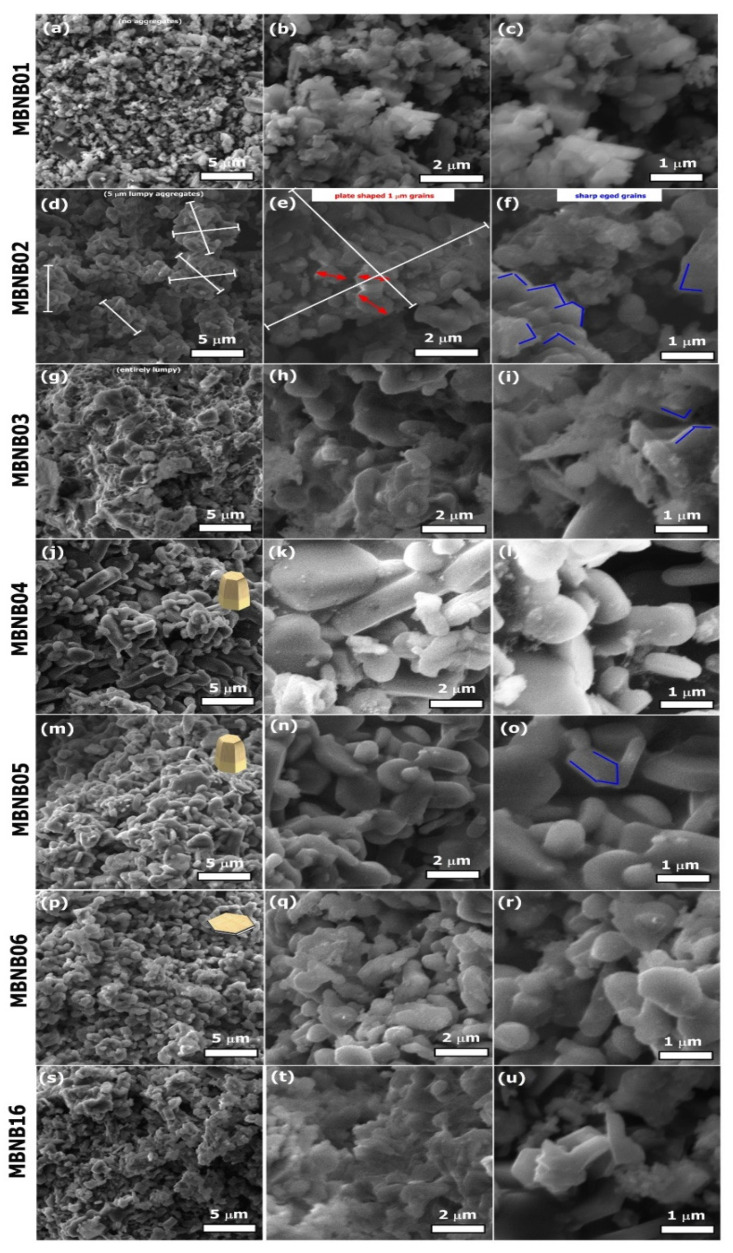
SEM images of the MgB_2_ powders. Images of each row correspond to the sample indicated on the left (see Table 1). The three columns correspond to a magnification of 10 k×, 30 k×, and 50 k×, respectively.

Concerning the material microstructure, a general SEM overview is shown in Figure 3. Sample MBNB02 has hexagonal-shaped plate-like grains about 1 μm in size with sharp edges and almost clear surfaces (see Figure 3f, blue contours). This hexagonal plate-like morphology of MgB_2_ grains is frequently reported in the literature [44,45,46]. However, on a larger scale, the same sample shows the abundant presence of lumpy spherical aggregates approximately 5 μm in diameter (see Figure 3d). One of these aggregates is highlighted with crossed markers in Figure 3e. It is plausible to assume that the 1 wt.% of unreacted Mg of this sample acts as an adhesive between MgB_2_ individual grains and facilitates aggregation, as already reported in the literature [47,48].

Table 1 shows that the unreacted Mg phase fraction increases to 11 wt.% for sample MBNB04 and subsequently further increases to an average value of 26 wt.% in the MBNB03, MBNB05, and MBNB16 samples, respectively. These values are more similar to the average value of about 18 wt.% for the unreacted Mg trapped between the MgB_2_ grainswhich has been typically reported for the Mg-RLI process [42]. Correspondingly, the MgB_2_ grain attachment is enhanced in these samples, as testified by the SEM images in Figure 3g–o.

Indeed, the most apparent microstructural feature in these images is the presence of non-porous lumpy aggregates that suppress the presence of sharp edges in the MgB_2_ grains, promoting the appearance of smooth surfaces and rounded vertexes and edges. Moreover, plate surfaces sometimes appear to be decorated with nanometric spherical entities.

From the point of view of the preparation process, the main difference is that for this set of samples (i.e., MBNB03, MBNB05, and MBNB16), amorphous B regions were somehow “gently manually pressed” in the reaction chamber, whereas in the case of sample MBNB02, only freely packed amorphous B was utilized. Sample MBNB04 lays in an intermediate situation because even if the B powder regions were “gently manually pressed”, the processing duration was prolonged to 4 h and some Mg loss occurred by leakage out of the reaction chamber, resulting in an unreacted Mg fraction of 11 wt.% only. For this sample, hexagonal prism-shaped 5 μm long grains could be observed, suggesting that Mg facilitated the attachment of the MgB_2_ hexagonal plates along the direction normal to the *ab*-plane, thereby resulting in a local *c*-axis oriented growth with an increased prism height due to the increased synthesis duration.

On the contrary, for the sample processed for 6 hours (MBNB06), XRD shows no unreacted Mg, and SEM observation reveals uniformly distributed 1 μm hexagonal plate-shaped MgB_2_ grains. Even if this sample was processed for a longer duration, no indication of aggregates could be detected, in contrast to sample MBNB02. Therefore, focusing our considerations on the samples already shown in Figure 1 and Figure 2, it is possible to note that the grain aggregation seems to decrease by gradually increasing the synthesis duration from 2 to 4 and 6 h.

**Figure 4:**
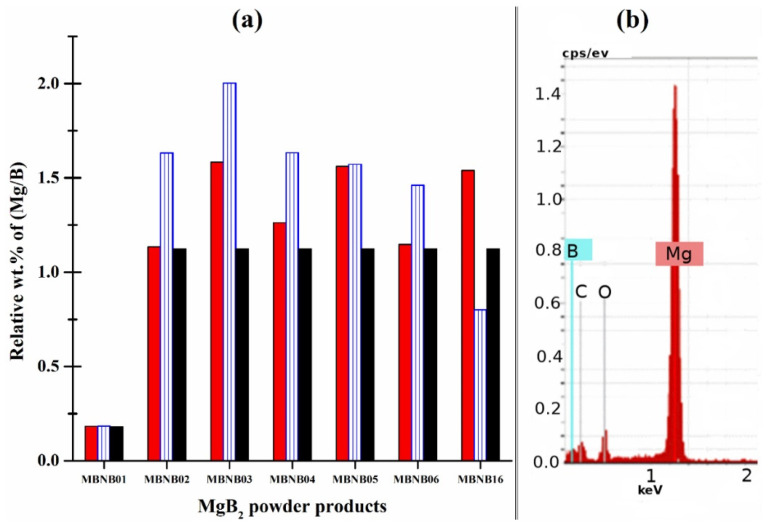
(**a**) Mg:B elemental ratio for the different samples expressed in terms of wt.%, as obtained from different analysis/calculation methods. Red and blue bars are calculated from XRD and EDX data, respectively. Black bars represent the expected values for a pure MgB_2_ (or Mg_2_B_25_, in case of sample MBNB01) stoichiometry; (**b**) EDX spectrum of a representative sample (MBNB06) with indications of the elemental peaks detected.

Figure 4b shows the typical spectrum measured by the EDX analysis, where the B, C, O, and Mg K_α_ characteristic peaks can be identified. It is well-known that the determination by EDX of the absolute wt.% of very light elements such as B, C, and O is highly demanding and requires special experimental and calibration procedures to achieve reliable results [49]. Therefore, these EDX results have to be considered semi-quantitative and useful for a comparison between our samples only. More specifically, C and O peaks can be ascribed to contaminations typically coming from either the sample holder carbon tape or from the grain surface and can be neglected for further analysis. If the EDX composition results are renormalized to 100% for the sum of Mg and B only, the data represented by the blue bars in Figure 4a can be obtained for the Mg:B ratio in terms of weight. These data should be compared to the expected theoretical values in the case of pure MgB_2_ (Mg:B = 1.12) or Mg_2_B_25_ (Mg:B = 0.18) phase, depending on the sample. These expected values are represented by the black bars of Figure 4a. In order to have a more accurate reference, the same Mg:B ratio in terms of wt.% has also been calculated from the XRD Rietveld refinements reported in Table 1. In this case, when MgO or unreacted Mg were present among the crystalline phases, these contributions to the total Mg amount were included in the calculation of the Mg:B weight ratio. These data are represented by the red bars in Figure 4a. As a first comment, it can be noticed that the only case where EDX, XRD, and theoretical determinations fully match is for the sample mainly consisting of Mg_2_B_25_ (MBNB01). Secondly, concerning the comparison between XRD determination and expected values, Figure 4a is useful to highlight the samples with a large content of unreacted Mg (where Mg:B ≈ 1.5) and distinguish them from the ones with almost pure phase MgB_2_ (Mg:B ≈ 1.1) or moderate unreacted Mg content (Mg:B ≈ 1.3). On the other hand, concerning the relationship between EDX and XRD determination, it is possible to see that most of the EDX values overtake the corresponding XRD ones. This can be explained simply by both the lower accuracy of EDX results and by the lower atomic number of B that favors the emission of Auger electrons over characteristic X-rays, leading to some underestimation of the B content with respect to Mg [50,51,52]. In this regard, the Mg:B weight ratio determined by XRD phase volumetric extraction can be considered more accurate than the surface-sensitive EDX measurement, which can just give a semi-quantitative confirmation of XRD results.

**Figure 5:**
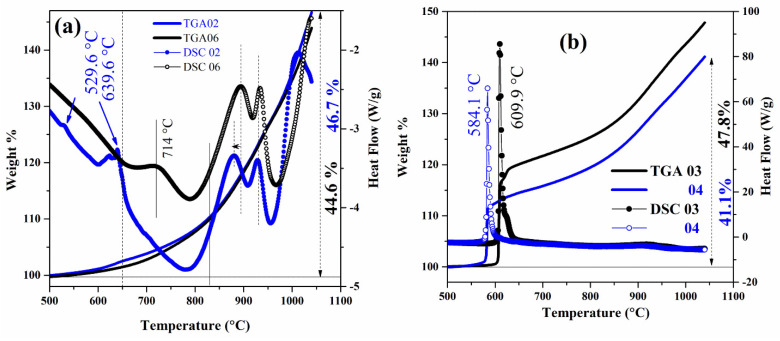
TG/DSC measurements in air of: (**a**) two representative samples consisting of almost pure MgB_2_ (MBNB02 and MBNB06); (**b**) two representative Mg-rich samples (MBNB03 and MBNB04). Thin solid lines refer to TG, and circles correspond to DSC curves. A single color (black or blue) in the same panel identifies the same sample.

Further insight into the sample composition and microstructure can be obtained by means of TG/DSC measurements. Figure 5 shows some examples of TG/DSC data in air for both almost pure MgB_2_ samples (panel (a)) and unreacted Mg-rich samples (panel (b)). It was reported in the past that pure MgB_2_ phase in the air starts its oxidation above 600 °C with the formation of a MgO film on its surface and then accelerates its weight gain at about 950 °C because of the occurrence of the decomposition reaction: 2MgB_2_ (*s*) → MgB_4_ (*s*) + Mg (*g*), with consequent oxidation of the gaseous Mg [53]. This behavior can be observed also in the TG data of Figure 5, with a total weight gain between 40% and 50% in the temperature range 500 °C–1050 °C, which is very similar to what was reported in the literature [53,54,55]. On top of this general trend, some additional phenomena can be detected and interpreted with the help of corresponding DSC thermal events. Indeed, in Figure 5b, two very sharp exothermic peaks about 60–80 W/g in height can be observed at temperatures of about 584 °C and 610 °C, which are accompanied by a correspondingly sudden increase in the weight by 10–15%. On the other hand, in Figure 5a, much smaller exothermic peaks (less than 1 W/g in height) are detected at a maximum temperature of about 640 °C, which correspond to a maximum weight gain of about 2.5%. Considering that the two almost pure MgB_2_ samples of Figure 5a contain a maximum of 2 wt.% of unreacted Mg, to be compared to the 11–29 wt.% of residual Mg in the two Mg-rich samples of Figure 5b (see Table 1), it is natural to ascribe all of these peaks to Mg thermal oxidation in air and to interpret the large difference in peak intensity in terms of the corresponding remarkable difference in Mg content. It should also be noticed that the temperatures at which these oxidation peaks can be observed (i.e., about 584 °C for the sample containing 11 wt.% Mg and 610 °C for the sample with 29 wt.% Mg) are much lower than the bulk Mg melting point (650 °C). This behavior has already been observed for Mg nanoparticles, whose surface undergoes oxidation processes at a temperature that decreases with decreasing the particle sizes, but in any case does not exceed 610 °C for particle sizes in the micron range [56,57]. Based on these observations, it can be assumed that the nanometric particles decorating the surfaces of the hexagonal MgB_2_ flake-shaped grains (see for instance (Figure 3k,l)) correspond to Mg nanoparticles undergoing low-temperature oxidation because of their size and because of a possible catalytic role played by MgB_2_ itself [58,59].

**Figure 6:**
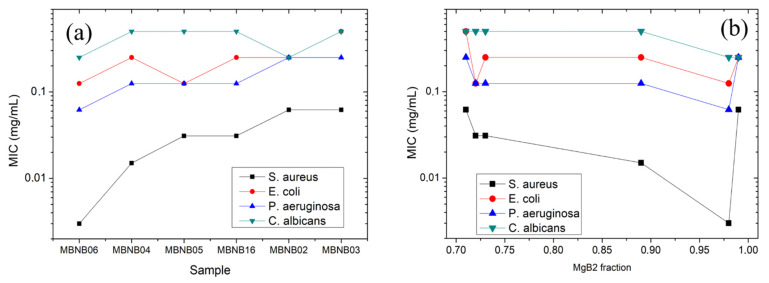
MIC values plotted as (**a**) a function of the overall sample antimicrobial activity (decreasing from left to right) and (**b**) as a function the MgB_2_ phase content listed in Table 1.

The MICs of the different samples against planktonic microbes are reported in Table 2, as determined via the microdilution method. It is apparent that the sample mainly consisting of Mg_2_B_25_ (MBNB01) does not show a remarkable antimicrobial activity, hindering a possible application in this direction for this compound. Instead, from the point of view of the microbial strains, it can be observed that all of the samples exhibit their lowest activity against *E. faecalis*, with MIC values not less than 0.5 mg/mL. The MIC values of the remaining four strains and six samples are shown in Figure 6. Panel (a) reports them in order of overall antimicrobial efficacy (decreasing from left to right), whereas in panel (b), they are shown as a function of the MgB_2_ phase content, as listed in Table 1.

It can be observed that, from a general point of view, in the range of concentrations we tested (i.e., from 0.5 to 0.0009 mg/mL), the highest antimicrobial activity is recorded against *S. aureus*, followed in order by *P.* *aeruginosa,* *E. coli,* and *C. albicans*. Moreover, with the partial exception of *C. albicans* only, it can be highlighted that all of the MIC values obtained for the present samples produced via the RLI method are lower than the ones recently reported in an analogous experiment performed using commercially available MgB_2_ powders [37]. In the specific case of *P. aeruginosa* (ATCC 27853), the RLI sample with the best antimicrobial activity shows an MIC value of 0.062 mg/mL that is 5 times lower than the corresponding best value (0.31 mg/mL) obtained for commercial powders. The best MIC value for RLI samples is as low as 0.003 mg/mL, which is achieved for sample MBNB06 against *S. aureus*. Figure 6b shows the same data as a function of the MgB_2_ phase wt.% of the samples. Although a general correlation can be observed between an increasing MgB_2_ content and decreasing MIC values, the notable exception of the sample with the highest MgB_2_ wt.% (MBNB02, MgB_2_ wt.% = 99) can be detected. According to Figure 3d, this sample has also shown a clear aggregation of the MgB_2_ grains in globules about 5 μm in size. This observation suggests that grain aggregation plays a role in reducing the MgB_2_ antimicrobial activity, probably due to the decrease in surface interactions with microbial cells, as already demonstrated for other materials such as alloys and metal oxide nanoparticles [60,61]. Indeed, many studies have shown that the contact between material and microbial surface plays a very important role in the antimicrobial activity, cumulated with other numerous factors, such as the surface charge, shape, presence of active oxygen, pH, physicochemical properties, microbial cell structure (Gram-positive versus Gram-negative bacteria, bacterial versus fungal wall), and microbial growth phase (planktonic versus biofilm) [62].

**Figure 7:**
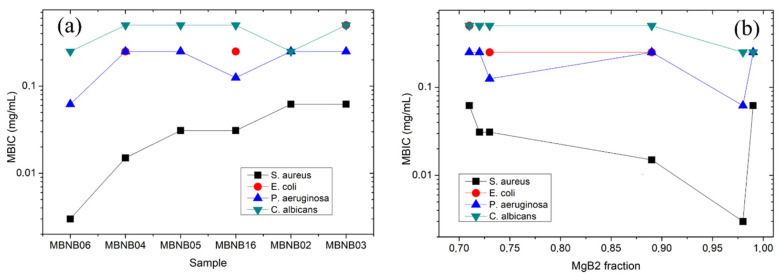
MBIC values plotted as (**a**) a function of the overall sample antimicrobial activity (decreasing from left to right) and (**b**) as a function of the MgB_2_ phase content listed in Table 1.

Concerning the antibiofilm activity, Table 3 reports the MBIC values for the different strains tested in our experiment. It is possible to observe that no antimicrobial activity is detected in the case of *E. faecalis* and very little effect is associated with sample MBNB01, confirming that Mg_2_B_25_ is not the best candidate for this kind of application. The data concerning the remaining samples and strains are plotted in Figure 7.

These data show that the MBIC values are exactly the same as the MIC ones for *S. aureus* and *C. albicans*, confirming the good activity of MgB_2_ against both kinds of bacterial growth [37]. Additionally, the MBIC and MIC values of *P. aeruginosa* are practically identical, with a small increase in MBIC for samples MBNB04 and MNB05 only (see Figure 7a). On the contrary, in the case of *E. coli* biofilms, the inhibitory activity of all of the samples is highly reduced, with most of the MBIC values laying out of the concentration range we have explored. When compared to the commercial powders, our results show a lower antimicrobial activity against *C. albicans* (by a factor of 2–3) and a higher one against *P. aeruginosa* (by the same factor) [37]. The relationship between the MBIC results and the MgB_2_ phase content (see Figure 7b) supports the existence of a general correlation between high MgB_2_ wt.% and low MBIC, but again with the exception of the sample with the highest MgB_2_ content, which confirms a significant role played by grain aggregation in determining the actual level of bioactivity.

Finally, Table 4 shows a comparison between the average MIC and MBIC values obtained for all of the tested strains. These data show that MIC values are less than or equal to the MBIC ones, but with a maximum difference limited to about 40% in the case of sample MBNB05, which confirms the very similar efficiency of the RLI powders against both planktonic and biofilm cells. This observation is very significant, especially taking into account that generally biofilms are more tolerant to higher concentrations of antimicrobials (which are often impossible to reach in vivo due to their toxicity) or that they require very complex therapeutic regimens, combining many antimicrobial substances [63,64].

## 4. Conclusions

We successfully produced MgB_2_ samples by means of the RLI method, exploring different Mg:B stoichiometric ratios, synthesis temperatures, and durations. Sample compositions were investigated by means of the XRD, EDX, and TG/DSC techniques, obtaining self-consistent and reliable results. MgB_2_ wt.% ranging from 2% to 99% were observed, with Mg, MgO, and Mg_2_B_25_ as the remaining components and with different levels of MgB_2_ grain aggregation. The bioactivity tests showed that the MIC values of the powders produced via RLI are generally better than the ones of commercially available powders, whereas in the case of biofilms, the MBIC values of the two classes are equivalent. The antimicrobial activity of the RLI powders appears to be correlated with the MgB_2_ wt.%, but grain aggregation also plays an important role. Therefore, further investigation is necessary to fully control the microstructure of MgB_2_ produced via RLI and to improve the effectiveness and reliability of its antimicrobial action.

## Figures and Tables

**Table 1:** Sample synthesis parameters (stoichiometric ratio Mg:B, temperature *T*, dwell time *t_d_*) and corresponding weight percentage fractions (wt.%) of the several crystalline phases (Mg_2_B_25,_ MgB_2,_ Mg, and MgO) detected in the samples. The first row corresponds to sample names. Final values of *R_wp_*, *R_exp,_* and GoF χ^2^ = *R_wp_*/*R_exp_* are also reported.

	MBNB01	MBNB02	MBNB03	MBNB04	MBNB05	MBNB06	MBNB16
Mg:B	1.4:2	1.4:2	1.8:2	1.4:2	1.4:2	1.4:2	1.4:2
*T* (°C)	670	800	800	800	760	800	800
*t_d_* (h)	2	2	4	4	6	6	2
Mg_2_B_25_	0.97	-	-	-	-	-	-
MgB_2_	0.02	0.99	0.71	0.89	0.72	0.98	0.73
Mg	0.01	0.01	0.29	0.11	0.28	-	0.22
MgO	-	-	-	-	-	0.02	0.05
*R_wp_*	6.48	9.45	9.58	19.18	17.51	9.13	9.97
*R_exp_*	1.79	5.67	5.72	15.81	13.98	5.44	6.75
GoF χ^2^	3.6	1.66	1.67	1.21	1.25	1.68	1.47

**Table 2:** Minimal Inhibitory Concentrations (MIC) of the samples expressed in mg/mL for the different microbial strains. Data are reported as mean ± SD.

	MBNB01	MBNB02	MBNB03	MBNB04	MBNB05	MBNB06	MBNB16	DMSO
*S. aureus*	>0.5	0.062 ± 0.002	0.062 ± 0.005	0.015 ± 0.001	0.031 ± 0.001	0.003 ± 0.001	0.031 ± 0.001	>50%
*E. faecalis*	>0.5	>0.5	0.50 ± 0.02	0.5 ± 0.1	0.50 ± 0.05	>0.5	>0.5	>50%
*E. coli*	>0.5	0.25 ± 0.05	0.50 ± 0.01	0.25 ± 0.04	0.125 ± 0.005	0.125 ± 0.003	0.25 ± 0.05	50%
*Ps. aeruginosa*	>0.5	0.25 ± 0.02	0.25 ± 0.05	0.125 ± 0.005	0.13 ± 0.05	0.062 ± 0.002	0.125 ± 0.005	50%
*C. albicans*	>0.5	0.25 ± 0.02	0.50 ± 0.03	0.50 ± 0.01	0.50 ± 0.01	0.25 ± 0.02	0.50 ± 0.01	50%

**Table 3:** Minimal Biofilm Inhibitory Concentration (MBIC) of the samples expressed in mg/mL for the different microbial strains. Data are reported as mean ± SD.

	MBNB01	MBNB02	MBNB03	MBNB04	MBNB05	MBNB06	MBNB16	DMSO
*S. aureus*	0.5	0.50 ± 0.05	0.062 ± 0.003	0.062 ± 0.005	0.015 ± 0.0001	0.031 ± 0.002	0.003 ± 0.002	>50%
*E. faecalis*	>0.5	>0.5	>0.5	>0.5	>0.5	>0.5	>0.5	>50%
*E. coli*	>0.5	>0.5	>0.5	0.5 ± 0.1	0.25 ± 0.05	>0.5	>0.5	50%
*Ps. aeruginosa*	0.5	0.50 ± 0.03	0.25 ± 0.05	0.25 ± 0.04	0.25 ± 0.10	0.25 ± 0.05	0.06 ± 0.04	25%
*C. albicans*	0.5	0.50 ± 0.05	0.25 ± 0.01	0.50 ± 0.06	0.50 ± 0.06	0.5 ± 0.1	0.25 ± 0.02	50%

**Table 4:** Comparative average MIC and MBIC values in mg/mL for the different powders. For each powder, the values are averaged over all of the five tested microbial strains. Data are reported as mean ± SD.

	MBNB01	MBNB02	MBNB03	MBNB04	MBNB05	MBNB06	MBNB16
MIC	0.50 ± 0.06	0.26 ± 0.15	0.36 ± 0.22	0.28 ± 0.06	0.26 ± 0.10	0.19 ± 0.03	0.28 ± 0.06
MBIC	0.5 ± 0.1	0.31 ± 0.05	0.36 ± 0.15	0.30 ± 0.07	0.36 ± 0.02	0.26 ± 0.05	0.28 ± 0.10

## Data Availability

Not applicable.

## Sample Availability

Samples of all of the compounds described in the paperare available from the authors upon reasonable request.
